# Pravastatin Prevents Aortic Atherosclerosis via Modulation of Signal Transduction and Activation of Transcription 3 (STAT3) to Attenuate Interleukin**-**6 (IL**-**6) Action in ApoE Knockout Mice

**DOI:** 10.3390/ijms9112253

**Published:** 2008-11-14

**Authors:** Xiaoxu Zhou, Dan Li, Wei Yan, Weimin Li

**Affiliations:** 1 Department of Cardiology, The First Affiliated Hospital of Harbin Medical University, 23 Youzheng Str, Nangang District, Harbin 150001, P.R. China. E-Mails: zhouxiaoxu@yahoo.com.cn (X. Z.); yanwei7777@yahoo.com.cn (W. Y.); 2 Eye hospital, The First Affiliated Hospital of Harbin Medical University, 23 Youzheng Str, Nangang District, Harbin 150001, P.R. China. E-Mail: ld_anny@hotmail.com (D. L.)

**Keywords:** Pravastatin, atherosclerosis, IL-6, STAT3, SOCS3

## Abstract

The purpose of this study was to determine whether pravastatin’s prevention of aortic atherosclerosis via attenuation of IL**-**6 action depends on modulation of STAT3 activity. Male apoE knockout (apoE-/-) mice fed on a diet containing 1.25% cholesterol (wt/wt) were divided into pravastatin group provided with pravastatin (80 mg kg^-1^ per day) and atherosclerosis group. After eight weeks, pravastatin significantly prevented atherosclerotic lesion and reduced levels of IL-6 in serum and lesion, and significantly decreased expressions of phosphorylated STAT3 (pSTAT3) and increased suppressor of cytokine signaling 3 (SOCS3) expressions in lesions. Our results suggested that pravastatin’s aortic atherosclerosis preventing action via attenuation of IL-6 action may partially depend on modulation of STAT3 activity.

## 1. Introduction

Atherosclerosis is the underlying disorder in the majority of patients with cardiovascular disease and is commonly considered to be an inflammatory vascular disease [1]. Recent research has established a key pathogenic role for several inflammatory mediators, such as IL-6, in all stages of this disease [2, 3]. Activation of IL-6 signal transduction involves gp130 dimerization [4], followed by tyrosine phosphorylation of STAT3. STAT3 is the primary mediator of the IL-6-Janus kinase (JAK)-STAT3 signaling pathway and is responsible for the nuclear actions of IL-6 [5]. The SOCS proteins are a growing family of suppressors of cytokine signaling molecules that are feedback inhibitors of cytokine signaling pathways [6]. The signaling of IL-6 can be inhibited by SOCS3 [7].

The local JAK-STAT3 pathway is important in controlling diverse pathways in the cardiovascular system [8, 9] and IL-6-JAK-STAT3 pathway could be activated in atherosclerosis [10]. To summarize, not only STAT intracellular signaling pathways can be associated with atherosclerosis, but STAT proteins also help explain new properties of therapeutic agents [11]. Therefore, a therapeutic strategy targeting STAT3 activity may be beneficial in treating atherosclerosis.

Pravastatin, a hydrophilic inhibitor of 3-hydroxy-3-methylglutaryl coenzyme A (HMG-CoA), has lipid-lowering activity and influences on main mechanisms of atherogenesis [12]. Pravastatin has a direct positive effect on plaque stability that is unrelated to lipid lowering. Improvements in plaque stability must have resulted from some other actions of pravastatin, an effect usually described as pleiotropism [13]. Recent works pointed to beneficial pleiotropic (nonlipid) effects of pravastatin include anti-inflammatory action [14, 15] and additional cholesterol-independent effects of statins on cellular signal transductions [16]. However, the relationship between pravastatin preventing aortic atherosclerosis and modulating STAT3 remains elusive.

To elucidate the cellular and molecular mechanisms involved in pravastatin prevention of atherosclerosis, we investigated the impact of pravastatin on STAT3 activity in combating atherosclerosis in apoE-/- mice.

## 2. Results and Discussion

### 2.1. Analysis of atherosclerotic lesions

**Figure 1.**
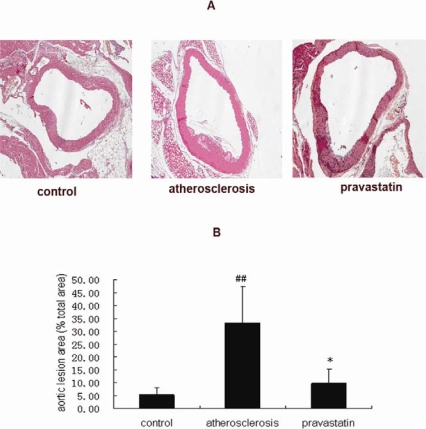
A. Representative photomicrographs show paraffin sections of atherosclerotic plaques from aortic roots of mice. Magnification 100X for Hematoxylin and Eosin staining. B. Ratio of the atherosclerotic lesion area compared to the total vessel wall area in mice. ^##^*P* <0.01 versus control mice; * *P*<0.05 versus atherosclerosis mice. (n=6 per group).

We determined the size of the atherosclerotic lesion in each group of the mice. In the present experiment, the mice in the atherosclerotic group showed atherosclerosis lesions in the aortic root, while the mice in the pravastatin group showed a marked reduction in aortic lesion size. (Figure 1A). Quantitative analysis of atherosclerotic area was expressed by the ratio of the lesion area versus the total vessel wall area. As shown in Figure 1B, the ratio in atherosclerotic mice (33.20%) was increased compared with that in control mice (5.32%) (P <0.01), on the other hand, the ratio in mice provided with pravastatin (9.65%) was lessened, compared with that in the atherosclerotic mice (P <0.05).

### 2.2. Analysis of serum lipid

As shown in Table 1, the serum levels of low density lipoprotein-cholesterol (LDL-C) (21.76±6.29 mmol L^−1^) and total cholesterol (TC) (26.59±7.25 mmol L^−1^) in atherosclerotic mice were significantly increased compared with that of LDL-C (0.27±0.12 mmol L**^–^**^1^) and TC (2.23±0.60 mmol L^−1^) in control mice (P < 0.01), however those concentrations of LDL-C (12.81±9.14 mmol L^−1^) and TC (17.20±11.67 mmol L^−1^) were not significantly different in mice provided with pravastatin, in contrast to atherosclerotic mice.

### 2.3. Concentrations of IL-6 in serum and aorta

**Figure 2.**
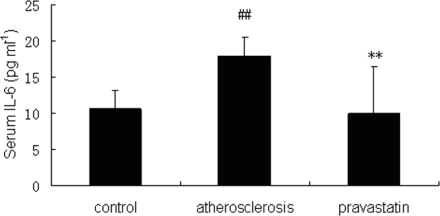
Concentrations of IL-6 (pg mL^−1^) in serum were measured by ELISA. ^##^*P*<0.01 versus control mice; ** *P*<0.01 versus atherosclerosis mice (n=6 per group).

**Figure 3.**
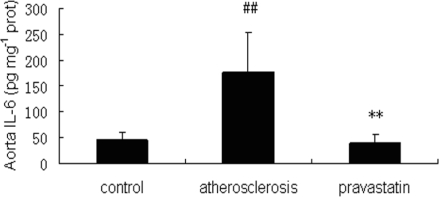
Concentrations of IL-6 (pg mg^−1^ protein) in thoracoabdominal aorta are measured by ELISA. ^##^*P*<0.01 versus control mice; ** *P*<0.01 versus atherosclerosis mice (n=6 per group).

Concentrations of IL-6 in serum and aorta were measured by ELISA. As is shown in Figures 2 and 3, concentrations of IL-6 in serum (17.99±2.51 pg mL^−1^) and aorta (176.04±77.62 pg mg^−1^ protein) of atherosclerotic mice were significantly increased compared with concentrations of IL-6 in serum (9.93±6.57 pg mL^−1^) and aorta (40.56±14.79 pg mg^−1^ protein) of control mice (P <0.01), but concentrations of IL-6 in serum (10.56±2.56 pg mL^−1^) and aorta (45.49±15.01 pg mg^−1^ protein) of mice provided with pravastatin were significantly decreased compared with IL-6 concentrations in serum and aorta of atherosclerotic mice (P <0.01)

### 2.4. Expression of pSTAT3 in aorta

**Figure 4.**
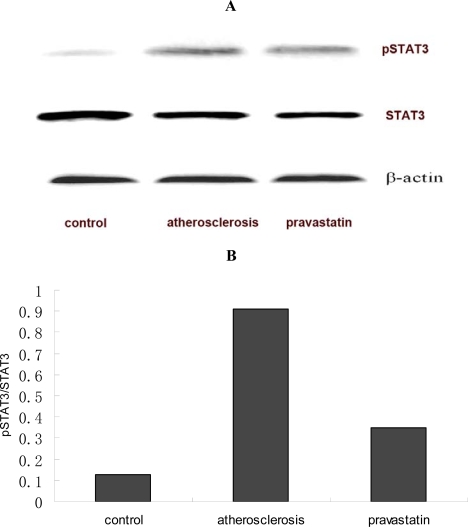
A. Expressions of pSTAT3 and STAT3 proteins extracted from the thoracoabdominal aortas of mice and examined by Western Blotting. B. The expression of pSTAT3 protein was quantified by MutiGauge V 3.0 Imager and normalized to STAT3 protein (n=6 per group).

It is well known that pSTAT3 acts on the active form of STAT3. The expression of pSTAT3 in thoracoabdominal aorta was examined by Western Blotting. As shown in Figure 4, the expression of pSTAT3 in atherosclerotic mice was significantly up-regulated compared with that observed in control mice, while that expression in mice provided with pravastatin were significantly down-regulated compared with that in atherosclerotic mice. Three similar experiments had similar results.

### 2.5. Expressions of SOCS3 in aorta

**Figure 5.**
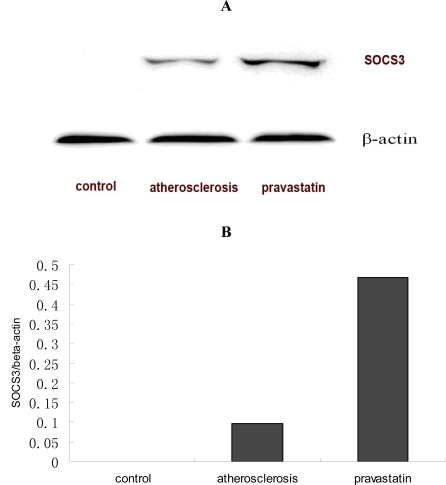
A. The expression of SOCS3 proteins extracted from the thoracoabdominal aortas and examined by western blotting. B. The expression of SOCS3 protein quantified by MutiGauge V 3.0 Imager and normalized to β-actin protein (n=6 per group).

SOCS3 can regulate STAT3 activity in a negative way. The expression of SOCS3 in thoracoabdominal aorta was examined by Western Blotting. As shown in Figure 5, the expression of SOCS3 in atherosclerotic mice were significantly attenuated compared with that seen in control mice, while simultaneously, that expression in mice provided with pravastatin was significantly heightened compared with that in atherosclerotic mice. Three similar experiments had similar results.

In the present study, the aortic atherosclerotic lesions in apoE-/- mice could be induced by a diet containing 1.25% cholesterol (wt/wt) and prevented by pravastatin. Our data indicates that pravastatin can prevent high-cholesterol diet-induced atherosclerosis in apoE-/- mice. The results are consistent with the current research in large clinical trials like CARE (Cholesterol and Recurrent Events) [17], WOSCOPS (West of Scotland Coronary Prevention Study) [18] and LIPID (Long-term Intervention with Pravastatin in Ischaemic Disease) [19].

Recently, HMG-CoA reductase inhibitors have been postulated to have pleiotropic effects, such as restoration of endothelial function, stabilization of atherosclerotic plaques, reduction of oxidative stress, anti-inflammatory actions, inhibition of thrombosis, and suppression of smooth muscle cell proliferation, improvement of insulin sensitivity and enhancement of vasculogenesis [12]. In our study, the results showed that the concentrations of TC and LDL-C in serum were significantly enhanced in atherosclerotic apoE-/- mice, however, the concentrations of TC and LDL-C were not significantly different in apoE-/- mice provided with pravastatin compared with atherosclerotic apoE-/- mice. Our results indicated that the effects of pravastatin on preventing atherosclerosis may not merely depend on decreasing serum lipid in apoE-/- mice. Previous research [20, 21] reported that pravastatin had no effect on plasma cholesterol concentration in apoE knockout mice and pravastatin treatment of atherosclerosis could not only be attributed to the reduction in plasma lipid levels, but also to its non-cholesterol properties [13]. Consequently, this established animal model is beneficial to the research on non-cholesterol lowering actions of pravastatin preventing atherosclerosis.

Cytokines are well known to participate in the process of atherogenesis. Therefore, inhibition of cytokine agents has beneficial effects on the prevention of atherosclerosis. Consistent with our results, recent studies suggest that IL-6 plays an important role in the process of atherosclerosis [1], which is attributable to the fact that high cholesterol-diets induce the formation and overexpression of oxidized low-density lipoproteins, instrumental in triggering the early atherosclerotic events, and seems to uphold the inflammatory environment in atherosclerosis [22], including increases of IL-6 levels. Our data show that compared with atherosclerotic apoE-/- mice, IL-6 concentrations in serum and aortic atherosclerotic lesions of apoE-/- mice provided with pravastatin were significantly decreased, indicating that pravastatin may prevent atherosclerosis partially by attenuating IL-6 secretion. The results were consistent with the study showing that pravastatin therapy has been shown to provide significant reduction in the levels of inflammatory markers [15]. HMG-CoA reductase catalyzes the conversion of HMG-CoA to mevalonate [23], thereby the inhibitory effect of pravastatin on IL-6 expression may be due to mevalonate starvation. However, the mechanisms of pravastatin attenuating IL-6 action during preventing aortic atherosclerosis are not yet fully understood. It is well known that identifying the signaling mechanisms that are common to many factors may eventually lead to the development of better therapeutic agents against atherosclerosis [24]. Since IL-6 can induce STAT3 activation and phosphorylation, therefore, our results also suggested that pravastatin could decrease the upstream factor of STAT3 action in preventing aortic atherosclerosis.

Interestingly, a number of atherosclerosis-related gene products were upregulated by STAT3, such as fibrinogen, angiotensinogen, monocyte chemotactic protein-1 (MCP-1), the LDL-receptor, and the adenosine triphosphate (ATP)-binding cassette transporter A1 [25]. Local activation of the IL-6-Jak-STAT3 pathway may control many steps in the process of vascular remodeling, monocyte recruitment and neointimal formation [10]. Thus, STAT3 may possibly serve as a therapeutic target and has thus garnered a great deal of interest. Although recent research has reported that statins could reduce IL-6-induced phosphorylation of STAT3 [26], it remains intangible whether STAT3 could play a key role in intervening with IL-6 action during the process of pravastatin prevention of aortic atherosclerosis. In our study, the expression of pSTAT3 in apoE-/- mice on a cholesterol-rich diet were obviously higher than that expression in control mice, while the overexpression of pSTAT3 was significantly down-regulated in apoE-/- mice with cholesterol-rich diet supplemented with pravastatin. Our data indicated that pravastatin may down-regulate STAT3 activity and stream nucleus transcription molecules and gene expressions of atheroma-associated factors. Thus, our results suggested that pravastatin may prevent aortic atherosclerosis, in the part, by modulating STAT3 activity to attenuate IL-6 action.

Since tamping the level of STAT3 activity would seem to represent a more judicious approach to block the development of atherosclerosis, the future use of the STAT3 specific inhibitors could be a new strategy to control atherosclerosis. It is known that cytokine response and the activation of STAT can be negatively regulated by the suppressor of cytokine signaling (SOCS) proteins [27]. Recent studies have revealed that SOCS3 determines the gene program activated by IL-6, and upregulated SOCS3 may help enhance a blockade of STAT3 signaling [28]. Based on these results, induction of SOCS is a therapeutic strategy for treating inflammatory diseases [29]. In our current study, that expression of SOCS3 was enhanced significantly in apoE-/- mice supplemented with pravastatin. Our data indicated that pravastatin enhanced SOCS3 expression in negative regulation of IL-6 action. Thus, our results suggested that pravastatin may prevent aortic atherosclerosis, in the part, by enhancing the inhibitory factors of STAT3 activity to decrease IL-6 action.

In summary, besides advantageous lipid lowering, pravastatin appears to exert a direct beneficial effect on plaque stability. Pravastatin may down-regulate STAT3 and up-regulate SOCS3 as the inhibitory factor of STAT3 activities, thus preventing aortic atherosclerosis by attenuating IL-6 action. The present research reported that reduction of IL-6 in atherosclerosis by statins is observed not only in apoE-/- mice but also in human [30], therefore inhibition of STAT3 activity by pravastatin may also help prevent inflammation-mediated atherosclerosis in human.

## 3. Experimental Section

### 3.1. Animal experiments

The apoE-/- mice can spontaneously develop atherosclerotic lesions on a standard chow diet and can develop advanced unstable atherosclerotic lesions in the brachiocephalic/innominate arteries that have many of the morphological features of advanced atherosclerotic lesions in humans, including intraplaque hemorrhage and rupture [20].

Male apoE-/- mice (8 weeks old, C57BL/6J background) were purchased from the Animal Center of Beijing Medical University (Beijing, P.R. China) and bred in the animal house of School of Medicine, Shanghai Jiao Tong University (Shanghai, P.R. China). Mice were maintained at a temperature 22±2°C and relative humidity 55 ± 15%-controlled room with a 12-h light and dark cycle and fed with food and tap water *ad libitum*. All procedures were approved by the Animal Care and Use Committee of Shanghai Jiao Tong University. Pravastatin was kindly provided by Sankyo Co. Ltd. (Japan).

Male C57BL/6J mice (8 weeks old) fed on a normal chow diet were provided with Phosphate Buffered Saline (PBS) by oral gavages (10 mL kg^−1^ per day) as control group. Male apoE-/- mice fed on a diet containing 1.25% cholesterol and 0% cholate (wt/wt) were divided into pravastatin group provided with pravastatin (80 mg kg^−1^, 10 mL kg^−1^, per day) suspended in PBS by oral gavages and atherosclerosis group provided with PBS by oral gavages (10 mL kg^−1^ per day) (n=12 per group). The pravastatin doses were chosen according to previous studies [31] and preliminary experiments in mice.

Eight weeks later, mice were euthanized after overnight fasting via anesthetic by intraperitoneal injection of sodium pentobarbital (1 mg kg^−1^). Blood samples were taken before animals were killed and centrifuged at 1,000g for 20 min. Serum of each animal was collected. The excised aortic roots were examined for detailed histological analysis (n = 6 per group). The excised thoracoabdominal aortas (from the beginning of the aortic arch, just after the aortic roots, to the iliac bifurcation) were used for protein analysis (n = 6 per group).

### 3.2. Assessing atherosclerosis lesion

The aortic roots were embedded in paraffin. Sequential 5-μm sections were cut through the aortic root. We assessed atherosclerosis from six sections separated by 50 μm from one another. Paraffin sections were stained with hematoxylin and eosin. The first section displayed the entire 3-aortic valve leaflets. Images from sections were observed under microscope (Axioskop 20, ZEISS, Germany) and collected by camera (AxioCan HRC, ZEISS, Germany). The size of atherosclerotic lesion in each section was evaluated using Leica Qwin Image Processing and Analysis Software (Leica Microsystems, Wetzlar). In order to estimate the relative lesion size, we calculated the ratio of the lesion area compared with the total vessel wall area and expressed the ratio as a percentage. For each animal, every six sections were examined (n=6 per group).

### 3.3. Analysis of serum lipid

Concentrations of TC and LDL-C in serum were measured by Biochemistry Analyzer (ADVIA 1650, Bayer, Tarrytown NY, USA) according to the manufacturer’s instructions, using individual serum samples from six mice in each group.

### 3.4. Western Blotting analysis

The thoracoabdominal aortas extraction, SDS-PAGE and Western Blotting analysis were performed as previously described [32]. Briefly, the thoracoabdominal aortas tissue extracted were prepared by homogenation in lysis buffer (50 mM Tris, pH 7.5, 150 mM sodium chloride, 1 mM phenylmethylsulfonyl fluoride, 1 mM sodium orthovanadate, 1% Nonidet P-40, 50 mM sodium fluoride, 10 μg/mL proteinase inhibitors mixture, 10% glycerol) at 4°C, followed by centrifugation at 16 000 rpm at 4°C for 10 min. After quantification of protein concentrations, the supernatants were mixed in Laemmli loading buffer, boiled for 4 min, and then subjected to Western blotting analysis. PVDF were blotted against primary antibodies at 4°C for 16 h, washed with 0.05% (vol/vol) Tween 20 in TBS (pH 7.4), and incubated with horseradish peroxidase-conjugated secondary antibodies for 45 min. Protein bands were visualized by the enhanced chemiluminescence reaction method (Applygen Technologies Inc., Beijing) (n=6 per group). Primary antibodies included anti-phospho-STAT3 (Tyr705), anti-SOCS3, and anti-β-actin antibody which were purchased from Santa Cruz Biotechnology (Santa Cruz, CA, USA).

### 3.5. Analysis of ELISA

Concentrations of IL-6 in thoracoabdominal aorta and serum were measured by enzyme linked immunosorbent assay (ELISA). Concentrations of IL-6 were measured by commercially available Mouse IL-6 ELISA Ready-SET-Go! (eBioscience co, San Diego, USA), according to the manufacturer’s instructions (eBioscience co, Diego, USA) (n=6 per group). Concentrations of IL-6 were quantified by Universal Microplate Reader (ELx800, BioTek Instruments Inc, Winooski, Vermont, USA).

### 3.6. Statistical analysis

Data are expressed as means±SD. To determine whether variations amongst different groups are statistically significant, data were analyzed by one-way analysis of variance ANOVA followed by unpaired Student’s t-test. The results obtained were reproducible between experiments. Each data point represents n=6. Individual statistical comparisons of paired data were evaluated with P <0.05 representing significance.

## 4. Conclusions

Our findings furnish a new mechanism through which pravastatin can prevent aortic atherosclerosis via attenuating IL-6 action by the modulation of STAT3 activity in apoE-/- mice. Our findings provide a new therapeutic approach for insight into clinical benefits of pravastatin and further insights into the intervening approaches of atherosclerosis. Our research may initiate rational and new therapeutic concepts for pravastatin in the future.

## Figures and Tables

**Table 1.** Concentrations of LDL-C and TC in mice serum.

Groups	Serum LDL-C (mmol L^−1^)	Serum TC (mmol L^−1^)
Control	0.27±0.12	2.23±0.60
Atherosclerosis	21.76±6.29 ^##^	26.59±7.25 ^##^
Pravastatin	12.81±9.14	17.20±11.67

Data are represented as means±SD (n=6).

## P<0.01 compared with control mice. (n=6 per group)
